# Principal component analysis of atrial fibrillation: Inclusion of posterior ECG leads does not improve correlation with left atrial activity

**DOI:** 10.1016/j.medengphy.2014.12.008

**Published:** 2015-02

**Authors:** Daniel Raine, Philip Langley, Ewen Shepherd, Stephen Lord, Stephen Murray, Alan Murray, John P. Bourke

**Affiliations:** a Department of Cardiology, Freeman Hospital, Newcastle Upon Tyne, UK; b School of Engineering, University of Hull, UK; c Institute of Cellular Medicine, Newcastle University, UK

**Keywords:** Surface electrocardiogram, Principal component analysis, QRST subtraction, Dominant frequency, Atrial fibrillation, ABS, average beat subtraction, AF, atrial fibrillation, CS, coronary sinus, DAF, dominant atrial fibrillation frequency, ECG, electrocardiogram, HRA, high right atrium, Hz, hertz, LA, left atrial, LVEF, left ventricular ejection fraction, LCPV, left common pulmonary vein, LLPV, left lower pulmonary vein, LUPV, left upper pulmonary vein, PCA, principal component analysis, PCs, principal components, PVs, pulmonary veins, RLPV, right lower pulmonary vein, RUPV, right upper pulmonary vein

## Abstract

**Background:**

Lead V_1_ is routinely analysed due to its large amplitude AF waveform. V_1_ correlates strongly with right atrial activity but only moderately with left atrial activity. Posterior lead V_9_ correlates strongest with left atrial activity.

**Aims:**

(1) To establish whether surface dominant AF frequency (DAF) calculated using principal component analysis (PCA) of a modified 12-lead ECG (including posterior leads) has a stronger correlation with left atrial activity compared to the standard ECG. (2) To assess the contribution of individual ECG leads to the AF principal component in both ECG configurations.

**Methods:**

Patients were assigned to modified or standard ECG groups. In the modified ECG, posterior leads V_8_ and V_9_ replaced V_4_ and V_6_. AF waveform was extracted from one-minute surface ECG recordings using PCA. Surface DAF was correlated with intracardiac DAF from the high right atrium (HRA), coronary sinus (CS) and pulmonary veins (PVs).

**Results:**

96 patients were studied. Surface DAF from the modified ECG did not have a stronger correlation with left atrial activity compared to the standard ECG. Both ECG configurations correlated strongly with HRA, CS and right PVs but only moderately with left PVs. V_1_ contributed most to the AF principal component in both ECG configurations.

## Highlights

• Individual posterior ECG leads better reflect left atrial activity compared to V_1_.
• Surface dominant AF frequency (DAF) calculated using principal component analysis.
• Modified 12-lead ECG (including posterior leads) compared to standard 12-lead ECG.
• Surface DAF from modified ECG did not correlate stronger with left atrial activity.
• Lead V_1_ dominant in AF principal component from both ECG configurations.

## 1 Introduction

It is widely accepted that the atrial waveform seen on the surface ECG during AF reflects intracardiac electrical activity [1,2]. The majority of research studies investigating surface ECG analysis in AF employ QRST subtraction as the method of extracting the AF waveform from the surface ECG. The two principal methods of QRST subtraction are average beat subtraction (ABS) and spatiotemporal QRST cancellation. ABS was initially developed to help identify P waves in ventricular tachycardia [3] and involves creation of an average QRST complex from a single ECG lead, which is then subtracted to leave the residual atrial signal [4,5]. It relies heavily on the assumption that the average QRST complex reflects each individual QRST complex accurately. However, QRST morphology can vary with the orientation of the heart's electrical axis and therefore, minor axis variations can result in significant QRST residuals appearing in the extracted AF waveform. Spatiotemporal QRST cancellation was developed to address this problem by using a multi-lead ECG (typically leads II, aVF and V_1_) to compensate for variations in electrical axis by transferring information between leads [6]. The majority of studies analysing the body surface AF waveform using these techniques have focused on lead V_1_ due to the relatively large amplitude AF waveform in this lead. This reliance on lead V_1_ detracts from the sensitivity of the results since V_1_ has been shown to correlate strongly with right atrial activity (*r* = 0.89) but only moderately with left atrial activity (*r* = 0.62) [2]. In the same study, posterior lead V_9_ had the strongest correlation with left atrial activity (*r* = 0.88) [2]. Therefore, results obtained using QRST subtraction of V_1_ will have an inherent right atrial bias and may not accurately reflect left atrial activity.

As an alternative to template subtraction methods, blind source separation techniques have been applied to extract atrial components from multi-lead recordings of AF by suppressing ventricular complexes [7–9]. The main advantage of these techniques is that they derive a ‘global’ AF waveform that has contributions from all ECG leads. One such method uses principal component analysis (PCA) [8,9].

Using a combination of PCA and Fourier analysis, we have previously shown that surface DAF is reproducible over time and changes appropriately with drug manipulation of the arrhythmia [9]. We have also demonstrated a reduction in surface DAF following creation of linear ablation lesions in the left atrium [10]. So far the focus of this work has been on the standard 12-lead ECG. However, given the evidence that posterior leads may provide a more accurate reflection of left atrial activity (the chamber responsible for the initiation and maintenance of AF in the majority of patients), the **aims** of this study were: (1) To establish whether surface DAF calculated using PCA of a modified 12-lead ECG (including posterior leads V_8_ and V_9_) had a stronger correlation with left atrial activity compared to the standard 12-lead ECG. (2) To assess the contribution of individual ECG leads to the AF principal component in both ECG configurations.

## 2 Materials

### 2.1 Patient recruitment and clinical characteristics

Study participants were recruited from patients with AF undergoing their first catheter ablation procedure for standard clinical indications. Class I and III antiarrhythmic drugs were discontinued five half-lives prior to ablation. Patients were excluded from the study if they were unable to give written informed consent or were taking amiodarone – because of its long half-life and effects on cardiac electrophysiology. The clinical characteristics of the 96 consecutive patients recruited are shown in Table 1. Their mean age was 57 ± 10 years and 79% were male. 54% had persistent AF and the mean AF history was 4 ± 4 years.

### 2.2 Study protocol

Surface ECG and intracardiac recordings were collected simultaneously on a Labsystem Pro™ recording system (Bard EP, C. R. Bard, Inc.) at a digital sampling rate of 1000Hz for offline analysis in Matlab^®^2011.

A 1-min recording was collected from patients in AF at the start of the procedure. For patients in sinus rhythm, AF induction was attempted using routine pacing manoeuvres. If AF was successfully induced, a 5-minute recording was collected to allow the arrhythmia to stabilise and AF parameters from the fifth minute were analysed. Patients were excluded from the study if AF could not be initiated or sustained for ≥5 minutes. Intracardiac recordings were collected from the coronary sinus (CS), high right atrium (HRA) and sequentially from each of the pulmonary vein ostia using a decapolar (Livewire™, St. Jude Medical, Inc.), quadpolar (Josephson, St. Jude Medical, Inc.) and bipolar irrigated-tip ablation catheter respectively. The distal poles of the CS catheter were positioned on the lateral aspect of the mitral valve ring, with proximal bipole CS_9__–__10_ just inside the ostium of the coronary sinus. The HRA catheter was positioned either in the right atrial appendage or high lateral right atrium depending on whether we were able to achieve a stable catheter position in the right atrial appendage.

## 3 Methods

### 3.1 Modified and standard surface 12-lead ECG measurements

Patients were assigned to modified or standard ECG groups depending on whether they were undergoing their AF ablation procedure with or without electroanatomical mapping guidance. The decision to use electroanatomical mapping was at the discretion of the physician and there were no significant differences in clinical characteristics between the two groups (Table 1). Positioning of the external reference patches for the CARTO^®^ 3 (Biosense Webster, Inc.) and Ensite Velocity™ (St. Jude Medical, Inc.) systems prevents optimum placement of posterior leads V_8_ and V_9_; therefore, the standard ECG configuration was used in these patients. In the modified ECG configuration, posterior leads V_8_ and V_9_ replaced leads V_4_ and V_6_. Unipolar ECG leads (aVR, aVL, aVF, V_1__–__6_, V_8_ and V_9_) were referenced to the Wilson Control Terminal as per the standard 12-lead ECG.

### 3.2 Surface AF waveform analysis

#### 3.2.1 AF waveform extraction – principal component analysis

The continuous AF waveform was extracted from the surface ECG using PCA as previously described by our group [9,10]. This is a multi-variable technique commonly used to identify and separate different sources in the data based on their degree of correlation. Mathematically it represents a linear transformation of the data to a new set of data variables (principal components (PCs)), which are uncorrelated.

The transformation is described by:

$$\begin{array}{c} PC_{1}=c_{1,1}l_{1}+c_{1,2}l_{2}\cdots c_{1,12}l_{12}\\ PC_{2}=c_{2,1}l_{1}+c_{2,2}l_{2}\cdots c_{2,12}l_{12}\\ \;\vdots \\ PC_{12}=c_{12,1}l_{1}+c_{12,2}l_{2}\cdots c_{12,12}l_{12} \end{array}$$

where *PC_i_* are the principal components, *l_j_* are the ECG leads and *c_i,j_* are the transform coefficients derived from the eigenvectors of the covariance matrix of the ECG leads arranged in order of descending eigenvalue. The transform coefficients describe the contribution of each lead to the PCs. In AF waveform analysis, the PCs contain the separated atrial, ventricular and noise components of the ECG signal. For subsequent AF waveform analysis, a single PC was identified visually as the one containing the largest amplitude AF waveform (*PC_AF_*). To quantify the contribution of each ECG lead to *PC_AF_*, we report the absolute value of the transform coefficients (|*c*_*AF, j*_|) separately for standard and modified 12-lead ECG configurations.

#### 3.2.2 AF waveform extraction – average beat QRST subtraction

As only one ECG configuration (standard or modified) was recorded in each patient, the AF waveform extracted from lead V_1_ using ABS (as described in [4,5]) was used as a control to allow comparisons of the strength of correlation between surface and intracardiac DAF measurements between the two ECG configurations.

#### 3.2.3 Intracardiac waveform analysis

PCA and Fourier analysis were used to calculate the intracardiac DAF from each bipole on the catheters positioned in the CS, HRA and pulmonary vein ostia. The most distal CS bipole (CS_1__–__2_) was excluded from the analysis because of the predominant ventricular activity and low amplitude atrial electrograms frequently recorded at this location.

##### 3.2.3.1 Dominant AF frequency

**Fig. 1:**
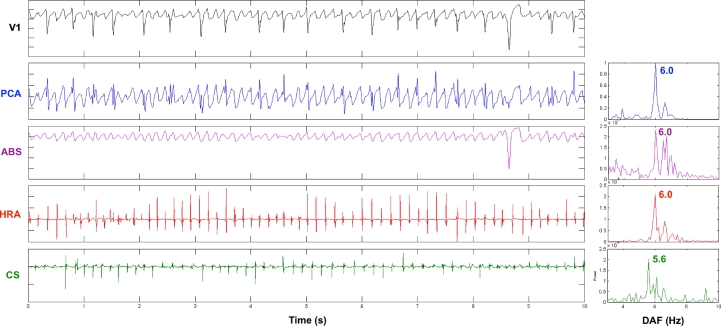
Ten-second section of ECG lead V_1_ with extracted AF waveform (PCA and ABS) and intracardiac recordings from HRA and CS. Corresponding frequency spectra and DAF are shown.

Power spectral density of the body surface and intracardiac atrial signals was performed by periodogram and the DAF was defined as the AF frequency with the highest power in the range 3–10 Hz [9,10]. Fig. 1 shows a 10-s section of ECG lead V_1_ with the extracted AF waveform (PCA and ABS) and intracardiac recordings from the HRA and CS. The corresponding frequency spectra and DAF are shown. Surface and intracardiac DAF were analysed in consecutive 10-s sections and mean values across the 1-min recordings are reported.

### 3.3 Statistical analyses

Continuous variables are expressed as mean ± SD. Patient characteristics were compared between the modified and standard ECG groups using Student's independent *t*-test for continuous variables and Pearson's chi-squared test for categorical variables. Surface DAF calculated using PCA and ABS from modified and standard ECG configurations was correlated with intracardiac DAF using Pearson's correlation. Fisher r-to-z transformation was used to evaluate the significance of the difference between comparable correlation coefficients. All tests were two-tailed and *p* < 0.05 was considered statistically significant.

## 4 Results

### 4.1 Modified vs. standard 12-lead ECG

Surface DAF from both modified and standard 12-lead ECG configurations correlated strongly with intracardiac DAF in the HRA, CS and right-sided pulmonary veins with all correlations being significant at the 0.01 level (Tables 2 and 3). Surface DAF from the standard but not the modified ECG correlated strongly with LUPV DAF (PCA *r* = 0.79; ABS *r* = 0.84, *p* < 0.01 vs. PCA and ABS *r* = 0.43, *p* < 0.05).

In addition, there was only moderate correlation between surface DAF from either ECG configuration and LLPV DAF (modified *r* = 0.43; standard *r* = 0.50, *p* < 0.05) (Tables 2 and 3). In the ABS control group, surface DAF from the standard ECG had a significantly stronger correlation with LUPV DAF compared to the modified ECG (0.84 vs. 0.43; *p* < 0.01) (Table 4).

In addition, surface DAF from the modified ECG had a stronger correlation with intracardiac DAF from HRA (0.91 vs. 0.82) and CS_5__–__6_ (0.81 vs. 0.62), although these did not reach statistical significance. Therefore, the only significant difference between modified and standard ECG configurations in the PCA group was observed with CS_7__–__8_ where the modified ECG had a stronger correlation with intracardiac DAF compared to the standard ECG (0.89 vs. 0.74; *p* = 0.03) (Table 4).

### 4.2 ECG lead contribution to AF principal component

**Fig. 2:**
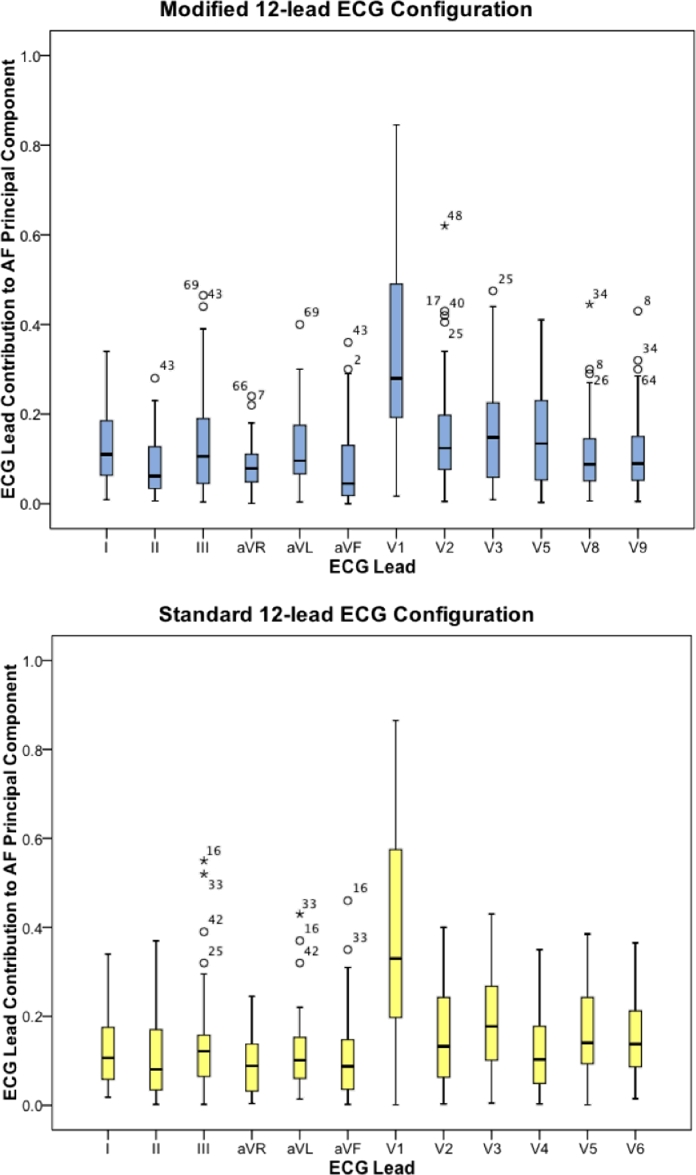
ECG lead contribution to AF principal component for modified and standard 12-lead configurations. Minimum, first quartile, median, third quartile and maximum principal component contribution are shown for each ECG lead. Outliers and extreme outliers are represented by circles and stars respectively and are labelled by case number.

We assessed the individual ECG lead contributions to the *PC_AF_* by plotting the absolute values of the transfer coefficients (|*c*_*AF, j*_|) for the modified and standard 12-lead ECG configurations (Fig. 2). Lead V_1_ contributed most to the *PC_AF_* in both ECG configurations, with a transfer coefficient typically three times that of the other leads. This is most likely a reflection of the large amplitude AF waveform typically seen in this lead.

## 5 Discussion

To our knowledge, this study is the first to correlate surface DAF measurements calculated using PCA and Fourier analysis of a modified 12-lead ECG configuration (including posterior leads V_8_ and V_9_) with intracardiac DAF measurements from the high right atrium, coronary sinus and pulmonary vein ostia. As we only recorded one ECG configuration (standard or modified) in each patient, we controlled for comparisons between the two by calculating surface DAF from lead V_1_ using ABS and Fourier analysis in all patients. Surface DAF from both modified and standard ECG configurations correlated strongly with intracardiac DAF from the high right atrium, coronary sinus and right-sided pulmonary veins. However, in general, there was only moderate correlation between surface DAF and intracardiac DAF from the left-sided pulmonary veins.

Taking into account the results from the average beat subtraction (ABS) control group, the only significant difference in the strength of correlation with intracardiac DAF between the modified and standard ECG configurations in the PCA group was in a single bipolar recording from the proximal part of the coronary sinus (CS_7__–__8_), which is usually more reflective of right atrial activity. Therefore, our results show that surface DAF calculated using PCA of a modified 12-lead ECG configuration (which includes posterior leads V_8_ and V_9_) does not have a stronger correlation with left atrial activity compared to the standard 12-lead ECG. This can be explained by the dominance of lead V_1_ in the AF principal component from both ECG configurations (Fig. 2) on account of its characteristic large amplitude AF waveform. This also explains the stronger correlation between surface DAF and intracardiac frequencies recorded from the right atrium and the strong correlation between surface DAF calculated using PCA (12-lead ECG) and ABS (lead V1) in both ECG configurations (modified: *r* = 0.91; standard: *r* = 0.86). The disparity between our results and the findings of Petrutiu et al. [2] can be explained by differences in the method used to extract the AF waveform from the surface ECG. They used QRST subtraction on individual ECG leads, whereas we used PCA on 12-lead ECG configurations. Whilst posterior lead V_9_ may have the strongest correlation with left atrial activity, its relatively small amplitude AF waveform ensures that it does not contribute significantly to the AF principal component in PCA.

## 6 Limitations

Firstly, we did not record both ECG configurations in each patient. However, we calculated surface DAF from lead V_1_ using ABS and Fourier analysis in all patients as a control measure to validate comparisons between the modified and standard ECG groups. Secondly, we only used recordings from the coronary sinus and pulmonary vein ostia to reflect left atrial activity. Previous studies have shown that the physical and electrical connections between the coronary sinus and left atrium can vary between patients [11,12] and so coronary sinus recordings may not reflect left atrial activity accurately in all cases [13].

Similarly, other areas of the left atrium such as the left atrial appendage, roof, septum and posterior wall commonly contain high frequency sites and were not sampled in this study. These sites may contribute to the surface DAF and therefore merit further study.

## 7 Conclusion

Surface DAF calculated using PCA and Fourier analysis of a modified 12-lead ECG configuration (which includes posterior leads V_8_ and V_9_) does not have a stronger correlation with left atrial activity when compared to the standard 12-lead ECG. Surface DAF from both modified and standard ECG configurations correlate strongly with right atrial activity, reflecting the dominance of lead V_1_ in the AF principal component.

## Ethical approval

This study complies with the Declaration of Helsinki and was granted a favourable ethical opinion by the National Research Ethics North West Committee (REC reference: 11/NW/0476). Written informed consent was obtained from all patients included in the study.

## Funding

This work was supported by the British Heart Foundation (Clinical Research Training Fellowship FS/11/47/28867 for D.R.), the National Institute for Health Research Newcastle Biomedical Research Centre (P.L. – no grant number) and the Northumberland, Tyne and Wear Comprehensive Local Research Network (J.P.B. – no grant number). The funding sources had no involvement in study design, data collection, analysis and interpretation, manuscript preparation and the decision to submit the article for publication.

## Conflict of interest

None declared.

## Figures and Tables

**Table 1:** Patient characteristics.

Characteristic	12-lead ECG configuration		*P* value
	Modified (*n* = 51)	Standard (*n* = 45)	
Age (years)	56.1 ± 11.1	58.2 ± 9.1	0.30
Male gender	40 (78%)	36 (80%)	1.00
Persistent AF	24 (46%)	28 (54%)	0.16
AF history (years)	4.0 ± 3.8	4.6 ± 4.8	0.54
LA volume (ml)	59.1 ± 20.3	60.6 ± 22.0	0.75
LVEF (%)	52.8 ± 5.8	53.1 ± 4.9	0.77
Hypertension	14 (28%)	21 (47%)	0.06
Diabetes	5 (10%)	3 (7%)	0.72

**Table 2:** Surface and intracardiac DAF correlation: Modified 12-lead ECG configuration.

12-lead ECG	Surface DAF	Surface DAF	Surface DAF	Intracardiac DAF (Hz)		*R* value	*R* value
configuration	(Hz) PCA	(Hz) ABS	*R* Value			PCA	ABS
Modified (*n* = 51)	6.52 ± 1.07	6.51 ± 1.13	0.91	HRA	6.54 ± 1.19	0.92^a^	0.91^a^
				CS_3__–__4_	6.04 ± 0.97	0.81^a^	0.78^a^
				CS_5__–__6_	6.11 ± 0.93	0.87^a^	0.81^a^
				CS_7__–__8_	6.06 ± 0.90	0.89^a^	0.83^a^
				CS_9__–__10_	6.01 ± 0.99	0.80^a^	0.76^a^
				LUPV	6.07 ± 0.87	0.43^b^	0.43^b^
				LLPV	6.10 ± 0.83	0.43^b^	0.43^b^
				LCPV	6.75 ± 0.83	0.88^a^	0.73^b^
				RUPV	5.90 ± 0.86	0.69^a^	0.62^a^
				RLPV	5.84 ± 1.00	0.72^a^	0.69^a^

a Correlation is significant at the 0.01 level (two-tailed).

b Correlation is significant at the 0.05 level (two-tailed).

**Table 3:** Surface and intracardiac DAF correlation: Standard 12-lead ECG configuration.

12-lead ECG	Surface DAF	Surface DAF	Surface DAF	Intracardiac DAF (Hz)		*R* value	*R* value
configuration	(Hz) PCA	(Hz) ABS	*R* value			PCA	ABS
Standard (*n* = 45)	6.13 ± 0.86	6.24 ± 0.65	0.86	HRA	6.28 ± 0.86	0.86^a^	0.82^a^
				CS_3__–__4_	5.69 ± 0.57	0.69^a^	0.68^a^
				CS_5__–__6_	5.66 ± 0.69	0.69^a^	0.62^a^
				CS_7__–__8_	5.65 ± 0.60	0.74^a^	0.73^a^
				CS_9__–__10_	5.70 ± 0.71	0.78^a^	0.78^a^
				LUPV	6.07 ± 0.80	0.79^a^	0.84^a^
				LLPV	6.06 ± 0.81	0.50^b^	0.50^b^
				RUPV	5.72 ± 0.64	0.62^a^	0.56^a^
				RLPV	5.60 ± 0.79	0.72^a^	0.71^a^

a Correlation is significant at the 0.01 level (two-tailed).

b Correlation is significant at the 0.05 level (two-tailed).

**Table 4:** Correlation coefficient comparison: Modified vs. Standard ECG configuration.

Intracardiac channel	PCA		*Z* value	*P* value	ABS		*Z* value	*P* value
	Modified ECG	Standard ECG			Modified ECG	Standard ECG		
	*R* value				*R* value			
HRA	0.92	0.86	1.33	0.18	0.91	0.82	1.70	0.09
CS_3__–__4_	0.81	0.69	1.23	0.22	0.78	0.68	0.99	0.32
CS_5__–__6_	0.87	0.69	2.18	0.03	0.81	0.62	1.82	0.07
CS_7__–__8_	0.89	0.74	2.19	0.03	0.83	0.73	1.15	0.25
CS_9__–__10_	0.80	0.78	0.21	0.83	0.76	0.78	−0.31	0.76
LUPV	0.43	0.79	−2.25	0.02	0.43	0.84	−2.80	<0.01
LLPV	0.43	0.50	−0.31	0.76	0.43	0.50	−0.31	0.76
RUPV	0.69	0.62	0.46	0.65	0.62	0.56	0.35	0.73
RLPV	0.72	0.72	0.00	1.00	0.69	0.71	−0.14	0.89

*Z* and *P* values were calculated using the Fisher r-to-z transformation.
